# Messenger Use and Video Calls as Correlates of Depressive and Anxiety Symptoms: Results From the Corona Health App Study of German Adults During the COVID-19 Pandemic

**DOI:** 10.2196/45530

**Published:** 2024-09-16

**Authors:** Johanna-Sophie Edler, Yannik Terhorst, Rüdiger Pryss, Harald Baumeister, Caroline Cohrdes

**Affiliations:** 1 Mental Health Research Unit Department of Epidemiology and Health Monitoring Robert Koch Institute Berlin Germany; 2 Department of Clinical Psychology and Psychotherapy Institute of Psychology and Education Ulm University Ulm Germany; 3 Department of Psychology Ludwig Maximilian University of Munich (LMU) Munich Germany; 4 Institute of Clinical Epidemiology and Biometry Würzburg University Würzburg Germany

**Keywords:** passive data, depression, anxiety, predicting mental health, mobile phone

## Abstract

**Background:**

Specialized studies have shown that smartphone-based social interaction data are predictors of depressive and anxiety symptoms. Moreover, at times during the COVID-19 pandemic, social interaction took place primarily remotely. To appropriately test these objective data for their added value for epidemiological research during the pandemic, it is necessary to include established predictors.

**Objective:**

Using a comprehensive model, we investigated the extent to which smartphone-based social interaction data contribute to the prediction of depressive and anxiety symptoms, while also taking into account well-established predictors and relevant pandemic-specific factors.

**Methods:**

We developed the Corona Health App and obtained participation from 490 Android smartphone users who agreed to allow us to collect smartphone-based social interaction data between July 2020 and February 2021. Using a cross-sectional design, we automatically collected data concerning average app use in terms of the categories *video calls and telephony*, *messenger use*, *social media use*, and *SMS text messaging use*, as well as pandemic-specific predictors and sociodemographic covariates. We statistically predicted depressive and anxiety symptoms using elastic net regression. To exclude overfitting, we used 10-fold cross-validation.

**Results:**

The amount of variance explained (*R*^2^) was 0.61 for the prediction of depressive symptoms and 0.57 for the prediction of anxiety symptoms. Of the smartphone-based social interaction data included, only messenger use proved to be a significant negative predictor of depressive and anxiety symptoms. Video calls were negative predictors only for depressive symptoms, and SMS text messaging use was a negative predictor only for anxiety symptoms.

**Conclusions:**

The results show the relevance of smartphone-based social interaction data in predicting depressive and anxiety symptoms. However, even taken together in the context of a comprehensive model with well-established predictors, the data only add a small amount of value.

## Introduction

### Background

Depression and anxiety are common disorders, with depression having a prevalence of 15.7% in Germany in 2017 before the COVID-19 pandemic [1,2]. In 2023, after the pandemic, approximately 19% of the adult population in Germany showed elevated rates of depressive symptoms; in addition, the burden of anxiety symptoms was notable for 12% to 15% of the German population in 2022 [3]. People with major depression show leading symptoms such as depressed mood, loss of interest, or lack of drive, whereas persistent excessive worry and restlessness concerning everyday issues are leading symptoms in people with general anxiety disorder. Both depression and anxiety often incur high individual and societal costs [4]. Accurate and real-time measurement of depressive and anxiety symptoms allows for the identification of current risk factors (eg, pandemic-related factors) [5] as well as the design of preventive and treatment measures. Through digitalization and the spread of smartphones, new possibilities have arisen for flexible, cost-effective, and high-quality data collection on symptoms of mental disorders such as depression and anxiety [6,7]; for example, smartphone data (use data and survey data) can be collected in real time using ecological momentary assessment methodology [8]. Technological advances have enabled passive collection of smartphone use data (without user intervention), reducing interviewer and recall bias. In addition, it is useful to record depressive symptoms via behavioral measurement using objective digital data. With behavioral measurement (such as smartphone use data), it is not necessary for users to be aware of the changes in their behavior. Persons with depressive or anxiety symptoms may only become aware of a behavior change when it reaches a certain level, whereas passive behavior measurement captures each slow change over time, regardless of user awareness; for example, in the context of depressive symptoms, possible behavioral changes such as reduced activity or social withdrawal could be an indicator of a change toward depressive symptoms. Previous studies have shown that depressive symptoms [9-14] and anxiety symptoms [10,11,15] correlate with various smartphone-based social interaction data, such as the duration of phone calls, Facebook use, the number of SMS text messages sent, or total smartphone use time. Furthermore, the high proportion of interpersonal communication that is conducted via mobile devices allows for the measurement of an indicator of social interaction. Specifically, the pandemic showed that perceived social connectedness improved when smartphone use increased, resulting in improved well-being [16]. Social interaction, as indicated by the use duration or frequency of various smartphone social media apps, has been identified as a significant predictor of depressive and anxiety symptoms [17,18]. While time spent on Facebook positively correlates with depressive symptoms [18], social smartphone use is a negative predictor [19]. Excessive smartphone use in general is positively associated with depressive symptoms [10]. Moreover, during the COVID-19 pandemic, daily face-to-face or video calls correlated with decreased depressive symptoms [20]. Anxiety symptoms are only positively related to consumption-related smartphone use and not to social interaction–related smartphone use [11].

Overall, studies analyzing smartphone data have mainly investigated isolated predictors and combinations of only a few predictors [21,22]. The question remains concerning the extent to which smartphone-based social interaction data will continue to a comprehensive model for the prediction of depressive and anxiety symptoms. Answering this question is of great importance for achieving an up-to-date, ecologically valid, and economic assessment of high-quality epidemiological data.

### Mental Health During the COVID-19 Pandemic

During the COVID-19 pandemic and the associated restrictions on social life, both the importance of digital social interaction and concerns about depressive and anxiety symptoms increased as the situation worsened [16,23]. Due to the dramatic increase in COVID-19 infections in Germany in March 2020, the German government implemented pandemic action plans to limit the spread of SARS-CoV-2, including quarantine measures, reduction of social contact, and restrictions on mobility. Over the following 18 months, social distancing measures were gradually eased, social contacts were restored, and restrictions on mobility were lifted, culminating in the reopening of shops, playgrounds, and schools in summer 2021 due to the decreased number of COVID-19 infections. In Germany, as in other countries, this period represented a balancing act between universal protection against a growing number of new COVID-19 infections and the preservation of individual psychological and physical health in the face of severe restrictions in daily life [23].

We mapped these pandemic-related changes in daily life in terms of internal and external aspects. Internal aspects refer to perceptions of the pandemic situation and resulting emotions such as fears and worries, which could be stressors. External aspects include changes in daily life due to pandemic control measures, which can also act as potential stressors.

Specifically, internal aspects within the scope of this study include the expected stigmatization of individuals with COVID-19 infection [24] and various related concerns; for example, previous research has revealed increased concerns about not receiving adequate medical care in case of COVID-19 infection due to lack of medical capacity [25] as well as concerns about infecting others with COVID-19 [24,25]. Pandemic-related social distancing also seemed to have altered social interaction, as suggested by reports of increased loneliness [26] and negative family climate [27,28]. Correspondingly, there is evidence showing an increase in psychosocial stress [29] and domestic violence during the pandemic [30].

Regarding external aspects, recent studies have discussed pandemic-related working conditions (eg, short-term work, reduced performance capacity due to home office work and home schooling of children, and increased workloads in the health sector) as stressful changes in daily routines [31-33].

In addition to these internal and external pandemic-specific changes, there are individual differences (reliable predictors existing before the pandemic) that may also be related to mental well-being during the pandemic and that could be considered stressors; for example, general health status should be considered a possible pandemic-related stress factor because this factor poses a potentially increased risk of severe disorder progression in the event of COVID-19 infection [34]. The same point applies to chronic conditions [30]. In addition, due to the pandemic, the availability of support services for treating mental disorders was temporarily limited, which placed a particular burden on people with preexisting diagnoses of mental disorders [35]. Finally, the risk of contracting COVID-19 infection should also be considered a potential stressor. The general population had to cope with this large number of possible stressors related to the COVID-19 pandemic and social restriction measures; therefore, individual coping strategies became increasingly important. In this context, early investigations in Germany showed an increase in adverse coping techniques, such as alcohol consumption [36]. Likewise, the restriction of sporting activities (in gyms or clubs) can be seen as a potential loss of a resource and method for adaptive coping [37].

According to the vulnerability-stress model [38], an accumulation of stressors is believed to increase the risk of depressive and anxiety symptoms. Quarantine and contact restriction measures result in a reduction in personal encounters, possible additional stress factors at work (eg, job loss and home schooling of children), and restrictions concerning personal lifestyle (eg, due to the closure of gyms and cinemas). Accordingly, the pandemic might have been a high-risk situation for the development or intensification of depressive and anxiety symptoms with the accumulation of multiple stressors in an individual [30,39]. In view of these findings, investigating depressive and anxiety symptoms during the COVID-19 pandemic may add new insights into the validity and predictive value of smartphone-based social interaction data. To date, the relative importance of such predictors in the context of a comprehensive model of established public health indicators has not yet been fully explained.

### Objectives of This Study

This study aimed to validate the predictive value of smartphone-based social interaction data in relation to depressive and anxiety symptoms via a comprehensive, data-driven approach, including well-established predictors (eg, chronic conditions and partnership status) from survey data collected during the COVID-19 pandemic. In many existing studies on the topic, smartphone data have been considered in isolation [21,22]. However, to evaluate these objective (smartphone-based social interaction) data as predictors, we opted for a data-based approach that integrates all reliable predictors into a holistic model and automatically identifies the optimal set of predictors. In this way, we were able to test the extent to which various smartphone-based social interaction parameters (eg, video calls and social media) contribute to the prediction of depressive and anxiety symptoms alongside, or instead of, empirically well-established predictors. Due to the ongoing COVID-19 pandemic during data collection, we also considered pandemic-specific factors (eg, COVID-19 infection and short-term work situations).

## Methods

### Sample and Procedure

The study is part of the Corona Health App project, a collaboration between the Robert Koch Institute, the University of Würzburg, the University of Ulm, and the University of Regensburg in Germany. The project includes an extensive cross-sectional app-based baseline survey, followed by a reduced weekly ongoing longitudinal survey for adults. The collection of both smartphone data and survey data was carried out via the Corona Health App, which is why it was necessary for participants to download and install the app from the Google Play Store or the Apple App Store to participate in the study. Recruitment was carried out through press releases by the participating institutions, media reports, and social networks. Before they took part in the study, participants were provided comprehensive information about the collection of select passive smartphone-based social interaction data and anonymized storage of the data; in addition, they were asked to provide informed consent. We collected passive smartphone-based social interaction data for 1 week before administering the questionnaire. Duration of use was recorded as the time during which the app was running in the foreground.

The cross-sectional analyses included 490 German residents who completed the baseline questionnaire between July 2020 and February 2021 and consented to the collection of smartphone-based social interaction data. During this period, Germany experienced a summer with very low COVID-19 infection numbers and eased restrictions on public life. In the autumn, a second wave of infections started, which worsened as autumn passed into winter and led to lockdown measures starting in mid-December. These measures continued until the end of the survey period for this study. Participants ranged in age from 18 to 78 (mean 42.46, SD 13.33) years, and the gender ratio was relatively balanced, with 52.9% (259/490) of the participants being women, 45.9% (225/490) being men, and 1.2% (6/490) being transgender individuals. Whereas 7.8% (38/490) of the participants had no school-leaving certificate or had lower than a secondary school certificate with or without training, 64.5% (316/490) had a technical college or university degree. A clinician-based lifetime diagnosis of mental disorder was reported by 43.7% (214/490) of the participants. As the recording of app use is not allowed on Apple devices, our reporting sample was limited to Android smartphone users, who, with a market share of 64.7% in Germany [40], constitute the clear majority of users. From the original sample of 1760 participants, 18 (1.02%) were excluded for failing the plausibility check, which assessed correspondence between similar items, straightlining, intraindividual response variability, and extreme outliers. Of the remaining 1742 participants, 1052 (60.39%) were Android smartphone users. Of these 1052 participants, 490 (46.58%) consented to the collection of data concerning social interaction app use. A detailed description of the sample and descriptive statistics of all variables can be found in Multimedia Appendix 1.

### Ethical Considerations

Ethics approval was granted by the ethics committee of the University of Würzburg (130/20-me). Furthermore, the app was developed in accordance with the regulations for medical devices. Participants were given comprehensive information about the type of data collected as well as the data collection, processing, and dissemination procedures and were asked to provide consent. Data storage was anonymized. The study protocol with details on the data collection, processing, and dissemination procedures has been described previously [41]. The study fulfils the criteria of the STROBE (Strengthening the Reporting of Observational Studies in Epidemiology) statement [42]. Participation was voluntary and not financially compensated.

### Measures

#### Depressive and Anxiety Symptoms

Depressive and anxiety symptoms were measured with the German version of the Patient Health Questionnaire, German version (PHQ-D) [43]. Depressive symptoms (9 items; eg, the stem question “Over the last week, how often have you been bothered by” followed by “little interest or pleasure in doing things?” “depression, melancholy, or hopelessness?” “difficulty falling asleep or staying asleep, or increased sleep?” “tiredness or feeling of having no energy?” “reduced appetite or excessive desire to eat?” “a bad opinion of yourself; feeling of being a failure or having disappointed the family?” “difficulties concentrating on something, eg, when reading the newspaper or watching television?” “thoughts that you would rather be dead or want to harm yourself?” as well as “Were your movements or speech so slowed down that others would notice? Or were you on the contrary ‘fidgety’ or restless and therefore had a stronger urge to move than usual?”) were rated on a 4-point scale: 0=*not at all*, 1=*on single days*, 2=*on more than half of the days*, and 3=*almost every day*. The 7 anxiety symptoms (eg, the stem question “Over the last week, how often have you been bothered by” followed by “not being able to stop or control worrying?” “nervousness, anxiety, or tension?” “excessive worries regarding various matters?” “difficulties to relax?” “restlessness, making it difficult to sit still?” “quick annoyance or irritability?” “a feeling of fear, as if something bad is going to happen?”) were answered on a 4-point scale: 0=*I haven’t been doing this at all*, 1=*I’ve been doing this a little bit*, 2=*I’ve been doing this a medium amount*, and 3=*I’ve been doing this a lot*. We summarized the answers for each scale as a total score ranging from 0 (minimum) to 27 (maximum) for depressive symptoms and 21 (maximum) for anxiety symptoms [44]. The Cronbach α values for the internal consistency of the scales for depressive and anxiety symptoms were 0.89 and 0.85, respectively.

#### Smartphone-Based Social Interaction Data

As smartphone-based indicators of social interaction, we included a selection of communication data for apps open in the foreground over the past 7 days (video calls, phone calls, social media, SMS text messaging use, and messenger apps) and total smartphone use duration. Use times were averaged on a weekly basis in minutes. The following selection of communication apps was included: Skype, Skype Messenger, Zoom, Facebook, Facebook Messenger, Instagram, Snapchat, WhatsApp, and Telegram. Skype and Zoom belong to the category *video calls*. Phone calls were recorded by the device-specific phone app and included as a separate category. Facebook, Instagram, and Snapchat were grouped into the category *social media*. *SMS text messaging* *use* was also captured via the device-specific app and included as a separate category. Facebook Messenger, WhatsApp, and Telegram were combined into the category *messenger*.

#### COVID-19–Related Factors

As pandemic-related variables, we made use of an in-house–developed item indicating COVID-19 status and differentiating between the 3 categories of *tested positive for COVID-19*, *tested positive for COVID-19 but already recovered*, and *did not test positive for COVID-19*. Other variables included were stigmatization because of (suspected) COVID-19 infection using a modified version of the inventory of subjective experience of stigmatization [45]. Two items each were collected as indicators of the expectation and actual experience of stigmatization. Expectation of stigmatization experience (eg, “Do you think people will value you less if they know that you are infected with COVID-19 or if you were?”) was scored on a 5-point scale ranging from 1=*always* to 5=*never*, and for the actual experience of stigmatization (eg, “Have you ever been teased, bullied, or harassed because you were infected with COVID-19, or have you seen such behavior toward others?”), participants answered on a dichotomous scale (yes or no). We calculated sum scores for the expectation and experience of stigmatization, and the Pearson *r* values for the interitem correlations were 0.41 and 0.43, respectively. Furthermore, we assessed pandemic-related concerns (concern about inadequate medical treatment in case of COVID-19 infection due to lack of medical capacity and concern about infecting others with COVID-19). The items were rated on a 3-point scale ranging from 0=*not bothered* to 2=*bothered a lot*. As employment status may have changed due to the measures implemented to combat the spread of SARS-CoV-2, participants answered several items that were developed in-house regarding employment status (ie, short-term work, unemployed, currently unable to pursue regular profession due to health protection measures, or on sick leave), wage loss (none, slight, or high), predominant workplace (home office, regular working location, or a combination of both), and employment as a health care professional (yes or no). Nominal categories were converted to dummy variables. We also used items developed in-house to ask about COVID-19 infections among relatives and friends (no, currently ill, or recovered). Those who reported COVID-19 infections in their social environment were asked whether they had lost relatives or friends to COVID-19 (no or yes). Both categorial variables were dummy coded and included in the analyses.

#### Reliable Predictors of Depressive and Anxiety Symptoms

As indicated by previous research, feelings of loneliness [46-48], psychosocial distress [49], family climate [50], physical abuse [51], general health status or chronic conditions [52], coping [53], alcohol consumption [54], and physical activity [55] are correlated with depressive or anxiety symptoms. We measured loneliness using the 3-item Socio-Economic Panel loneliness scale [56]. The answers were rated on a 5-point scale ranging from 1=*very often* to 5=*never*. An example item was “How often do you feel left out?” We calculated a sum score, and the Cronbach α value for internal consistency was 0.79. Family climate was assessed using 2 different items developed in-house: the actual climate rated on a 5-point scale ranging from 0=*very bad* to 4=*very good* and the perceived change in family climate since the beginning of the COVID-19 pandemic answered using 1 of 3 options: 0=*yes, it got worse*; 1=*no, it remained unchanged*; and 2=*yes, it improved*. Aspects of psychosocial distress were addressed by the PHQ-D stress module [57], comprising 10 items that were answered on a 3-point scale ranging from 0=*not bothered* to 2=*bothered a lot*. An example item was “In the past week, how much have you been bothered by stress at work outside of the home or at school?” We measured physical abuse using the PHQ-D [57] and an additional item developed in-house. The first item asked about experience of physical abuse in the past week, and the second item asked about experience of abuse approximately 12 months previously (yes or no). General health status was assessed using the first 3 items of the Minimum European Health Module (self-perceived health, chronic conditions, and long-term activity limitation) [58]. Moreover, participants reported a clinician-based lifetime diagnosis of a mental disorder (yes or no). Situational coping strategies (during the COVID-19 pandemic) were measured using the Coping Orientation to Problems Experienced Inventory (Brief-COPE) [59]. This instrument comprises a total of 28 items assessed on a 4-point scale ranging from 1=*I haven’t been doing this at all* to 4=*I’ve been doing this a lot*. As a result of an exploratory factor analysis and a confirmatory factor analysis, the 4 coping factors were formed in accordance with the categories *problem-focused coping* (eg, “I’ve been taking action to try to make the situation better”), *support-focused* *coping* (eg, “I’ve been getting help and advice from other people”), *escape-avoidant*–*focused coping* (eg, “I’ve been using alcohol or other drugs to make myself feel better”), and *meaning-focused* *coping* (eg, “I’ve been trying to see things in a different light, to make things seem more positive”) [60] following previous studies [60-62]. We summed the respective items and calculated a mean score for the 4 subscales problem-focused coping, support-focused coping, escape-avoidant–focused coping, and meaning-focused coping. The Cronbach α values for the internal consistency of the 4 subscales were 0.77, 0.84, 0.69, and 0.74, respectively. In addition, we explored the frequency of alcohol consumption per week with an item developed in-house that was answered on a 5-point scale ranging from 0=*none* to 4=*six to seven times a week*. Moreover, we included an item developed in-house to indicate levels of physical activity during the past week that was answered on a 5-point scale ranging from 1=*no sporting activity* to 5=*regularly more than 4 hours per week*. We assessed personality with the German version of the Big Five Inventory-10 [63], which was answered on a 5-point scale ranging from 1=*disagree strongly* to 5=*agree strongly*. In line with the Big Five Inventory-10 scoring manual [63], we constructed 5 subscales with 2 items each for the dimensions of openness (eg, active imagination), conscientiousness (eg, doing a thorough job), extraversion (eg, being outgoing and sociable), agreeableness (eg, being generally trusting), and neuroticism (eg, easily becoming nervous). The Pearson *r* values for the interitem correlations of the 5 subscales were 0.31, 0.26, 0.60, 0.07, and 0.41, respectively. The level of education was determined by the general school-leaving certificate and the highest vocational qualification. These 2 areas were combined into 3 levels according to the Comparative Analysis of Social Mobility in Industrial Nations classification [64]. The first level includes the range from no school-leaving certificate to a lower secondary school certificate, the second level includes the range from a secondary school certificate to a general qualification for university entrance or a general or subject-linked higher education entrance qualification, and the third level includes technical school certificates and university degrees. We included each category as a dummy-coded variable. In addition, household size and the number of children were included as sociodemographic variables, each as a metric variable. We asked about marital status and living environment, specifically about the type of housing (house or apartment; with garden, with balcony, or with terrace). Multiple choices were allowed for this variable, and the response categories were integrated into the analyses, each as a separate dummy variable. We also included the participant’s age as a continuous predictor and the participant’s gender based on the 3 categories *woman, man,* and *transgender*. Categorical variables were included as dummy-coded variables in the analyses.

### Data Analyses

To analyze the predictive value of smartphone-based social interaction data for depressive and anxiety symptoms, we opted for a data-driven approach, using ridge, least absolute shrinkage and selection operator (LASSO), and elastic net regression [65]. Hyperparameter optimization was implemented using a grid search. These analyses allow us to perform a statistics-based variable selection [66] and answer the question concerning the extent to which each smartphone-based social interaction parameter explains a relevant proportion of the variance or whether the parameter is automatically removed from the model as statistically irrelevant. In addition to the smartphone-based social interaction data, we considered other relevant and well-established predictors of depressive and anxiety symptoms (eg, coping strategies and physical activity) as well as pandemic-related factors (eg, concerns and stigmatization). To avoid overfitting, we split the data set into training and test sets. Within the training set, we performed 10-fold cross-validation with 5-fold repetition [65]. The model was then estimated in the training set, and the outcome variable was estimated (ie, predicted mathematically) based on this model in the test set to validate the model. Here, the term *prediction* refers to a statistical procedure and not a temporal prediction in the future. The model with the best fit in terms of estimating depressive and anxiety symptoms was chosen based on indicators of the amount of measurement error (primarily the root mean square error [RMSE] and secondarily the mean absolute error [MAE]) as well as the amount of variance explained (*R*^2^). The predictive value was indicated for each significant predictor by means of the variable importance.

For the majority of questions, answers were required (forced choice); there were only 3 missing values in the data, all of which were related to items on the PHQ-9 scale. However, a precondition for the use of ridge, LASSO, and elastic net regression is a data set without missing values [67]. For this reason, missing data were imputed by single imputation with 500 iterations using the classification and regression trees method with the R package *mice* [68]. The continuous predictors were *z*-standardized, and the outcome variables remained in the original scaling.

## Results

### Overview

For the prediction of depressive symptoms, the model with the smallest MAE (3.04) was the LASSO regression (Table 1). The smallest RMSE (3.85) resulted from the elastic net regression, which also had the highest amount of variance explained (*R*^2^=0.61). Accordingly, the elastic net regression proved to be the model with the best fit, reducing the data set from 76 to 28 significant variables.

The model with the best fit for predicting anxiety symptoms was also an elastic net regression (MAE=2.56, RMSE=3.22, *R*^2^=0.57) and contained 30 significant predictors of anxiety symptoms (Table 2).

**Table 1:** Model fit indices for predicting depressive symptoms resulting from stepwise, ridge, least absolute shrinkage and selection operator (LASSO), and elastic net regression based on 50 replicate samples.

			Values, median (IQR; minimum-maximum)		Values, mean
**Mean absolute error**					
	Linear model	3.299 (3.050-3.519; 2.665-4.222)		3.295	
	Ridge	3.133 (2.922-3.315; 2.511-3.797)		3.135	
	LASSO	2.973 (2.845-3.204; 2.617-3.812)		3.055	
	*Elastic net^a^*	*2.998 (2.813-3.189; 2.553-3.855)*		*3.045*	
**Root mean square error**					
	Linear model	4.088 (3.851-4.407; 3.346-5.145)		4.146	
	Ridge	3.940 (3.708-4.201; 3.220-4.663)		3.967	
	LASSO	3.787 (3.590-4.060; 3.245-4.672)		3.856	
	*Elastic net^a^*	*3.772 (3.594-4.060; 3.286-4.680)*		*3.849*	
***R^2^***					
	Linear model	0.558 (0.524-0.636; 0.265-0.713)		0.562	
	Ridge	0.587 (0.529-0.656; 0.300-0.776)		0.586	
	LASSO	0.619 (0.553-0.677; 0.265-0.819)		0.612	
	*Elastic net^a^*	*0.619 (0.554-0.676; 0.264-0.817)*		*0.612*	

^a^The best model fit by parameter is highlighted in italics in each case; overall, elastic net regression has the best model fit due to the largest amount of variance explained (*R²*) and the smallest mean absolute error and root mean square error.

**Table 2:** Model fit indices for predicting anxiety symptoms resulting from stepwise, ridge, least absolute shrinkage and selection operator (LASSO), and elastic net regression based on 50 replicate samples.

		Values, median (IQR; minimum-maximum)	Values, mean
**Mean absolute error**			
	Linear model	2.683 (2.486-2.958; 1.917-3.534)	2.711
	Ridge	2.608 (2.409-2.834; 1.866-3.235)	2.614
	LASSO	2.605 (2.432-2.759; 1.814-3.120)	2.582
	*Elastic net^a^*	*2.560 (2.402-2.745; 1.749-3.130)*	*2.564*
**Root mean square error**			
	Linear model	3.395 (3.133-3.712; 2.438-4.395)	3.405
	Ridge	3.273 (3.002-3.551; 2.442-3.939)	3.277
	LASSO	3.319 (2.989-3.443; 2.344-3.873)	3.246
	*Elastic net^a^*	*3.283 (3.004-3.409; 2.309-3.776)*	*3.216*
***R^2^***			
	*Linear model^a^*	*0.522 (0.450-0.610; 0.312-0.767)*	*0.528*
	Ridge	0.536 (0.481-0.630; 0.366-0.787)	0.550
	LASSO	0.561 (0.491-0.606; 0.359-0.814)	0.563
	Elastic net	0.564 (0.498-0.625; 0.388-0.820)	0.569

^a^The best model fit by parameter is highlighted in italics in each case; overall, elastic net regression has the best model fit due to the largest amount of variance explained (*R²*) and the smallest mean absolute error and root mean square error.

### Smartphone-Based Social Interaction Data

Of the smartphone-based social interaction data included, only average weekly messenger use turned out to be a significant negative predictor of depressive and anxiety symptoms. The average weekly duration of video calls was a significant negative predictor only of depressive symptoms. In the model for the prediction of anxiety symptoms, the weekly average duration of SMS text messaging use remained a relevant negative predictor. The other smartphone-based social interaction parameters (weekly averaged use duration of phone calls and social media, as well as weekly averaged total smartphone use duration) were omitted from the best fit model.

### Predictors of Depressive and Anxiety Symptoms

The final models overlap in terms of the following 19 predictors (in order of importance according to the model for depressive symptomatology): escape-avoidant–focused coping, loneliness, good current family climate, poor general health status, a clinician-based lifetime diagnosis of mental disorder, age, neuroticism, conscientiousness, expectation of no stigmatization due to (suspected) COVID-19 infection, agreeableness, meaning-focused coping, a chronic condition, wage loss, inability to pursue current occupation due to health protection measures, being a transgender individual, average weekly messenger use, no relatives or friends lost to COVID-19, household size, and concern about not receiving adequate medical care in case of COVID-19 infection due to lack of medical capacity.

**Figure 1:**
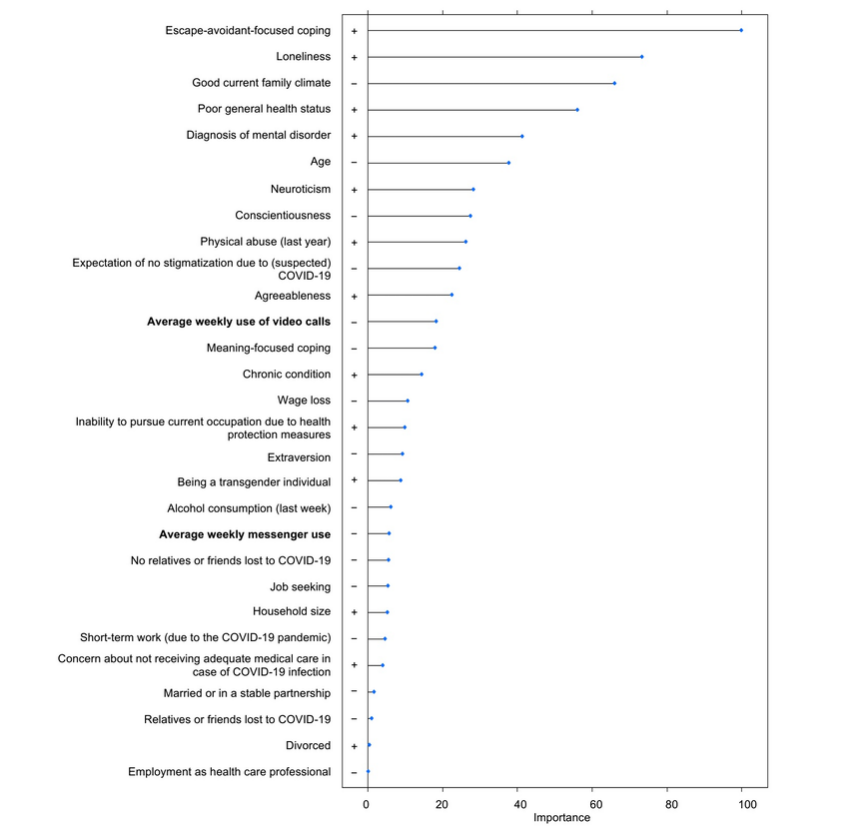
Total variable importance as an indicator of the contribution to the reduction of the estimation error in the prediction of depressive symptoms.

The following predictors were found to be exclusively relevant for the prediction of depressive symptoms (in decreasing order of importance): physical abuse, extraversion, alcohol consumption, job seeking, short-term work (due to the COVID-19 pandemic), being married or in a stable relationship, divorce, and employment as a health care professional. Figure 1 presents the order and directions of unique correlates.

**Figure 2:**
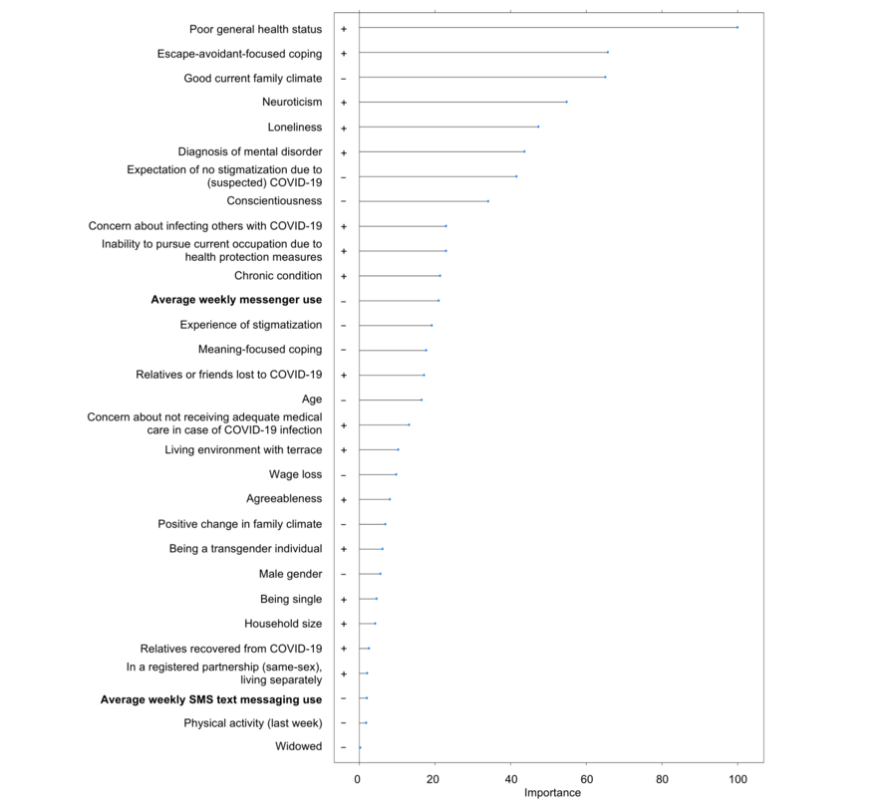
Total variable importance as an indicator of the contribution to the reduction of the estimation error in the prediction of anxiety symptoms.

By contrast, the following predictors proved to be exclusively relevant for anxiety symptoms (in decreasing order of importance): concern about infecting others with COVID-19, experience of stigmatization, a living environment with a terrace, being male, being single, having relatives who recovered from COVID-19, being in a registered (same-sex) partnership but living separately, average weekly SMS text messaging use, physical activity, and being widowed. The order and directions of unique correlates are presented in Figure 2.

## Discussion

### Principal Findings

This study investigated the prediction of depressive and anxiety symptoms on the basis of smartphone-based social interaction data supplemented by a comprehensive set of well-established predictors, while also taking into account relevant pandemic-specific factors. The results show that when well-established predictors are considered, only the average weekly duration of messenger use as a negative predictor adds significant value to the prediction of both depressive and anxiety symptoms. In only 1 of the 2 prediction models, the average weekly duration of video calls (depressive symptoms) and SMS text messaging use (anxiety symptoms) remained relevant.

These findings are based on data collected during the COVID-19 pandemic and underscore the importance of messenger, video call, and SMS text messaging use for maintaining social contact. This interpretation aligns with the fact that social contact is considered a preventive factor for depressive and anxiety symptoms in general [69,70] as well as during the COVID-19 pandemic in particular, as already discussed by several studies that have reported an increase in digital face-to-face interactions [71]. This point is particularly relevant in view of the social distancing measures imposed to reduce COVID-19 infection rates because in times of lockdown, digital interactions were sometimes the only means to achieve face-to-face contact and group meetings [16]. It is also conceivable that as depressive symptoms increase, accompanied by social withdrawal, smartphone-based interaction is also reduced.

In contrast to previous findings [21,22], other smartphone-based social interaction parameters (weekly averaged use duration of phone calls and social media, as well as weekly averaged total smartphone use duration) did not contribute to the prediction of depressive or anxiety symptoms in the context of the COVID-19 pandemic. One possible explanation is that prior studies examined individual or only a few nonsmartphone-based social interaction parameters; for example, loneliness is a well-established correlate of depressive symptoms [46-48]. Consistent with previously published results from this study [72] and other research [73], certain social interaction parameters, such as messenger use, are negatively correlated with loneliness. Thus, if loneliness is not taken into consideration, various smartphone parameters might seem to be significant correlates of depressive symptoms through spurious correlation. To avoid this effect, we included a comprehensive set of reliable predictors to provide insights into the additional value of smartphone-based social interaction data as predictors of depressive and anxiety symptoms. Future studies should also explicitly investigate the predictive value of smartphone-based social interaction data in isolation because these data are particularly attractive and accessible from a research economic point of view.

Another aspect that should be considered is the poor resolution of smartphone-based social interaction data. Whereas some studies have analyzed individual use patterns or changes over time [15,74], we decided to use a comprehensive but coarse-grained model and thus were unable to take into consideration more detailed information on, for example, content, motivation, or satisfaction with interactions. However, it has been found that the correlation between (social) media use and the symptoms of mental disorders varies according to use motives [75] and apps used [76]. A more detailed high-resolution analysis might help clarify associations between smartphone-based social interaction data and the symptoms of mental disorders in more detail.

Moreover, by taking into account a large number of proven predictors of depressive and anxiety symptoms, the results highlight the relevance of pandemic-related changes as additional predictors. The pandemic-related variables that remained significant in both our final models can be divided into internal aspects (cognitive appraisal processes and emotions) and external aspects (changes in daily life due to pandemic control measures).

The remaining internal aspects include expected stigmatization due to (suspected) COVID-19 infection and concern about not receiving adequate medical care in case of COVID-19 infection due to lack of medical capacity. In addition, the pandemic may have exacerbated the impact of other internal aspects of social interaction on feelings of loneliness [26] and family climate [27], which were already considered risk factors for the development of depressive and anxiety symptoms before the COVID-19 pandemic [46-48]. Correspondingly, loneliness and family climate were among the most important predictors of depressive and anxiety symptoms in our research. This result seems understandable if we view loneliness as an expression of an unmet need for attachment, which might increase the risk for developing depressive or anxiety symptoms. Similarly, social withdrawal can be an aspect of depressive symptoms and lead to family problems. By contrast, a subjective feeling of social connection prevents depressive symptoms. Family climate could be an indicator of the number of conflicts or tasks to be managed and thus an additional stressor that favors the development of psychopathological symptoms. Similarly, having a positive family climate as a resource could prevent the development of psychopathological symptoms.

The external aspect experienced in everyday life that predicts symptoms of depression and anxiety is the inability to pursue one’s current occupation due to health protection measures (eg, due to the closure or suspension of work activities). With this study, we were able to replicate previous findings in a more comprehensive statistical model that also included passive smartphone-based social interaction data [24-26]. The fact that the accumulation of stressors during the COVID-19 pandemic, along with the accompanying changes and limitations, can lead to an increase in depressive and anxiety symptoms was discussed [29,77]. Our data also showed high wage loss as a positive predictor of both depressive and anxiety symptoms, whereas a stable income seemed to correlate negatively with the development of psychopathological symptoms.

In contrast to other studies [78], our data indicated that losing relatives or friends to COVID-19 was a negative predictor of depressive and anxiety symptoms. In line with previous research findings, general health status, such as individual differences, chronic conditions, and a lifetime diagnosis of a mental disorder, showed particular relevance in relation to depressive and anxiety symptoms [29,30]. In addition, relations may have become even stronger in the context of the COVID-19 pandemic [79] because treatment services for people with both physical and mental health problems were temporarily reduced due to contact restrictions [80,81]. At the same time, willingness to seek help decreased, presumably due to concerns about contracting COVID-19 infection [79,82]. It can also be assumed that persons with poor physical health status have an increased risk of severe illness if infected with COVID-19 [34], possibly leading to increased health concerns that favor depressive and anxiety symptoms [83].

Coping strategies also played a role in predicting depressive and anxiety symptoms [53]. In line with previous findings [84], our results showed a positive association of escape-avoidant–focused coping with depressive and anxiety symptoms as well as a negative association with meaning-focused coping. The fact that coping strategies are gaining relevance is particularly understandable in view of the COVID-19 pandemic [53]: it is only when major stress arises in one’s life that the functionality and benefits of one’s coping strategies become evident; for example, Fullana et al [84] were able to show that reduced consumption of news concerning COVID-19 and pursuing hobbies were negatively related to depressive symptoms.

In addition, the personality facets of conscientiousness, agreeableness, and neuroticism proved to be significant predictors of depressive and anxiety symptoms, consistent with previous findings [85]. Due to the pandemic-related dissolution of work boundaries, it is conceivable that conscientiousness may have become even more relevant; for example, mandatory work from home shifted the responsibility for organizing work time entirely to the employee [86]. In this context, conscientiousness is particularly valuable for meeting professional requirements as well as managing tasks such as home schooling children or caring for underage children. By contrast, neuroticism was a positive predictor of depressive and anxiety symptoms, which is to be expected [87-89] because neuroticism’s core facet is trait anxiety [90]. In contrast to other research [87,88,91], our data showed that agreeableness was a positive predictor of depressive and anxiety symptoms.

Consistent with previous research [92], we found that older age was associated with lower depressive and anxiety symptoms. Younger age, already a risk factor for the development of depressive symptoms [93], seemed to play a particularly important role in the pandemic [92]. One of the reasons discussed is that younger people are much more connected via media and thus exposed to the news, which could have acted as a stressor during the COVID-19 pandemic and in the context of related news coverage [94]. Again, the task of analyzing this interaction effect remains an area for future studies. Another reason discussed is the greater vulnerability to stress and depressive symptoms associated with younger age [95]. If one also considers the restrictions in the everyday life of young people during the COVID-19 pandemic, it is noticeable that meeting friends, partying, dancing, attending vocational school in person, and collective studying suddenly disappeared due to contact restrictions. Accordingly, there was evidence of stressors such as feelings of loneliness and social isolation, especially among young adults [96]. Household size also proved to be a positive predictor of depressive and anxiety symptoms. This result is in line with previous findings that have discussed the fact that shared living space is associated with increased stress, depending on the number of residents [97].

The results provide initial indications that, taking established predictors into account, depressive and anxiety symptoms can be predicted using smartphone-based social interaction data. This represents an important step toward a future in which the early recognition of depressive symptoms via smartphones may become feasible. Even today, automated feedback based on smartphone-based social interaction data could enable early preventive health-promoting measures tailored to individual results.

### Strengths and Limitations

A major strength of this study is the comprehensive set of data comprising smartphone-based social interaction app use data as well as survey data. These data allowed us to create a holistic model of mental health predictors during the COVID-19 pandemic. Moreover, big data methodology was used, which allowed us to investigate the value of passive smartphone-based social interaction parameters for the prediction of depressive or anxiety symptoms while taking into account a broad set of other well-established relevant factors. This approach made it possible to show that passive smartphone-based social interaction data contribute to the explanation of variance beyond established predictors, which emphasizes the relevance of these new data. Furthermore, by collecting data via smartphones, a large number of participants could be reached and interviewed despite contact restrictions.

A limitation of the study is the lack of representativeness of the data. The group with a high level of education and younger participants are overrepresented. In addition, the cross-sectional correlational study design is a limitation because it is not possible to investigate predictive direction for a large number of variables that are unlikely to be antecedents of depressive or anxiety symptoms (eg, household, age, and gender). The latter variables include all smartphone-based interaction data and a large share of putative internal predictors. The inclusion of established strong predictors (eg, chronic condition and partnership status) may have limited the explanatory power of the model with respect to weaker risk factors (eg, smartphone-based social interaction data). The collection of various sorts of smartphone-based social interaction data also presents a methodological challenge because many apps can be used to communicate in different ways (eg, written messages or video calls). Thus, the mode of communication cannot be clearly ascertained. Due to a lack of comparative data before the pandemic, potential changes over time could also not be taken into account.

### Conclusions

This study indicates that data concerning the time spent engaging in smartphone-based social interaction adds only limited value regarding the prediction of depressive and anxiety symptoms among German adults during the COVID-19 pandemic. Although predictive direction cannot be established, these results are in line with models of the etiology of depression and anxiety.

## Appendix

Multimedia Appendix 1 Descriptive statistics of the sample of German-speaking adults (n=490) in total and grouped by gender.

## Abbreviations

Brief-COPE: Coping Orientation to Problems Experienced Inventory
LASSO: least absolute shrinkage and selection operator
MAE: mean absolute error
PHQ-D: Patient Health Questionnaire, German version
RMSE: root mean square error
STROBE: Strengthening the Reporting of Observational Studies in Epidemiology

## Data Availability

The data sets generated and analyzed during this study are not publicly available because the participants’ informed consent did not cover consent for making the data publicly available but are available from the corresponding author on reasonable request.
