# Supplementary material for: Messenger Use and Video Calls as Correlates of Depressive and Anxiety Symptoms: Results From the Corona Health App Study of German Adults During the COVID-19 Pandemic
Source: J Med Internet Res. 2024 Sep 16;26:e45530. doi: 10.2196/45530 (PMC11443235; doi:10.2196/45530)
Supplement: Multimedia Appendix 1 [file jmir_v26i1e45530_app1.docx]

**Table 1**

Descriptive statistics of the present sample of N=490 German speaking adults in total and grouped by sex

|  | *Total* | | | | | | | | | *Female* | | | | | | | | | *Male* | | | | | | | | | *Divers* | | | | | | | | |
| --- | --- | --- | --- | --- | --- | --- | --- | --- | --- | --- | --- | --- | --- | --- | --- | --- | --- | --- | --- | --- | --- | --- | --- | --- | --- | --- | --- | --- | --- | --- | --- | --- | --- | --- | --- | --- |
| Variables | *N* = 490 | | | | | | | | | *n* = 259 (53 %) | | | | | | | | | *n =* 225 (46 %) | | | | | | | | | *n = 6 (1%)* | | | | | | | | |
|  | *M* | | *(SD)* | | *Median* | | *Min.* | | *Max.* | *M* | *(SD)* | | *Median* | | | *Min.* | | *Max.* | *M* | *(SD)* | | *Median* | | | *Min.* | | *Max.* | *M* | *(SD)* | | *Median* | | | *Min.* | | *Max.* |
| Smartphone use ^a^ |  | |  | |  | |  | |  |  |  | |  | | |  | |  |  |  | |  | | |  | |  |  |  | |  | | |  | |  |
| videocalls | 2.01 | | 4.50 | | | 1 | | 1 | 32 | 2.03 | | 4.56 | | 1 | | | 1 | 31 | 2.01 | | 4.50 | | 1 | | | 1 | 32 | 1 | | 0 | | 1 | | | 1 | 1 |
| phone calls | 33.15 | | 52.69 | | | 1 | | 1 | 178 | 28.50 | | 49.11 | | 1 | | | 1 | 175 | 38.75 | | 56.17 | | 1 | | | 1 | 178 | 23.67 | | 55.52 | | 1 | | | 1 | 137 |
| social media | 100.01 | | 103.83 | | | 67.5 | | 1 | 312 | 105.49 | | 107.35 | | 74 | | | 1 | 312 | 94.39 | | 99.33 | | 62 | | | 1 | 307 | 74.67 | | 117.93 | | 1 | | | 1 | 269 |
| messaging | 90.71 | | 98.51 | | | 52.5 | | 1 | 297 | 94.32 | | 98.80 | | 58 | | | 1 | 297 | 88.10 | | 98.87 | | 44 | | | 1 | 296 | 32.67 | | 49.55 | | 1 | | | 1 | 107 |
| messenger | 223.06 | | 140.68 | | | 222.5 | | 1 | 467 | 221.33 | | 134.64 | | 219 | | | 1 | 467 | 226.45 | | 147.69 | | 228 | | | 1 | 466 | 170.67 | | 137.66 | | 144 | | | 39 | 338 |
| total smart-  phone use | 245.5 | | 141.59 | | | 245.50 | | 1 | 490 | 238.63 | | 143.53 | | 222 | | | 2 | 489 | 253.50 | | 139.77 | | 261 | | | 1 | 490 | 242.33 | | 131.10 | | 273 | | | 43 | 384 |
| Stigmatisation |  | |  | |  | |  | |  |  |  | |  | | |  | |  |  |  | |  | | |  | |  |  |  | |  | | |  | |  |
| expectation ^b^ | 6.24 | | 1.77 | | 6 | | 2 | | 10 | 6.33 | 1.70 | | 6 | | | 2 | | 10 | 6.14 | 1.85 | | 6 | | | 2 | | 10 | 6 | 2 | | 6 | | | 3 | | 9 |
| experience ^c^ | 1.86 | | 0.43 | | 2 | | 0 | | 2 | 1.87 | 0.40 | | 2 | | | 0 | | 2 | 1.85 | 0.46 | | 2 | | | 0 | | 2 | 2 | 0 | | 2 | | | 2 | | 2 |
| Concerns ^d^ |  | |  | |  | |  | |  |  |  | |  | | |  | |  |  |  | |  | | |  | |  |  |  | |  | | |  | |  |
| lack of medical  capacity | 0.71 | | 0.74 | | 1 | | 0 | | 2 | 0.73 | 0.74 | | 1 | | | 0 | | 2 | 0.69 | 0.76 | | 1 | | | 0 | | 2 | 0.5 | 0.84 | | 0 | | | 0 | | 2 |
| to infect  someone | 1.33 | | 0.74 | | 1 | | 0 | | 2 | 1.46 | 0.69 | | 2 | | | 0 | | 2 | 1.18 | 0.78 | | 1 | | | 0 | | 2 | 1.33 | 0.52 | | 1 | | | 1 | | 2 |
| contracting  COVID-19 | 1.19 | | 0.73 | | 1 | | 0 | | 2 | 1.25 | 0.72 | | 1 | | | 0 | | 2 | 1.10 | 0.74 | | 1 | | | 0 | | 2 | 1.67 | 0.52 | | 2 | | | 1 | | 2 |
| getting  seriously  ill in case of  COVID-19  infection | 1.13 | | 0.75 | | 1 | | 0 | | 2 | 1.15 | 0.77 | | 1 | | | 0 | | 2 | 1.09 | 0.73 | | 1 | | | 0 | | 2 | 1.67 | 0.52 | | 2 | | | 1 | | 2 |
| Loneliness ^e^ | 8.81 | | 3.10 | | 9 | | 3 | | 15 | 9.09 | 3.00 | | 9 | | | 3 | | 15 | 8.54 | 3.18 | | 8 | | | 3 | | 15 | 6.67 | 3.50 | | 5 | | | 3 | | 12 |
| Family climate |  | |  | |  | |  | |  |  |  | |  | | |  | |  |  |  | |  | | |  | |  |  |  | |  | | |  | |  |
| currently ^f^ | 2.54 | | 0.84 | | 3 | | 0 | | 4 | 2.51 | 0.81 | | 3 | | | 0 | | 4 | 2.58 | 0.86 | | 3 | | | 0 | | 4 | 2.67 | 1.03 | | 3 | | | 1 | | 4 |
| change ^g^ | 0.64 | | 0.63 | | 1 | | 0 | | 2 | 0.65 | 0.66 | | 1 | | | 0 | | 2 | 0.63 | 0.60 | | 1 | | | 0 | | 2 | 0.83 | 0.75 | | 1 | | | 0 | | 2 |
| Psychosocial  distress ^h^ | 7.89 | | 4.69 | | 7 | | 0 | | 20 | 8.53 | 4.64 | | 8 | | | 0 | | 20 | 7.17 | 4.68 | | 6 | | | 0 | | 18 | 7.33 | 4.03 | | 6.5 | | | 3 | | 13 |
| General health  status ^i^ | 2.26 | | 0.85 | | 2 | | 1 | | 5 | 2.26 | 0.84 | | 2 | | | 1 | | 5 | 2.24 | 0.85 | | 2 | | | 1 | | 5 | 3 | 1.10 | | 3 | | | 1 | | 4 |
| Coping ^j^ |  | |  | |  | |  | |  |  |  | |  | | |  | |  |  |  | |  | | |  | |  |  |  | |  | | |  | |  |
| escape-avoi-  dant-focused | 3.45 | | 2.90 | | 3 | | 1 | | 16 | 3.55 | 2.85 | | 3 | | | 1 | | 16 | 3.32 | 2.92 | | 2 | | | 1 | | 14 | 4 | 4.29 | | 1.5 | | | 1 | | 16 |
| meaning-  focused | 9.40 | | 3.63 | | 10 | | 1 | | 19 | 10.13 | 3.63 | | 10 | | | 1 | | 19 | 9.10 | 3.59 | | 9 | | | 1 | | 19 | 8.5 | 2.07 | | 8 | | | 6 | | 11 |
| problem-focused | 9.91 | | 3.70 | | 10 | | 1 | | 19 | 10.53 | 3.45 | | 10 | | | 1 | | 19 | 9.19 | 3.86 | | 9 | | | 1 | | 19 | 10.67 | 3.61 | | 11 | | | 5 | | 16 |
| Support-focused | 1.86 | | 0.43 | | 2 | | 0 | | 2 | 1.87 | 0.40 | | 2 | | | 0 | | 2 | 1.85 | 0.46 | | 2 | | | 0 | | 2 | 2 | 0 | | 2 | | | 2 | | 2 |
| Alcohol  consumption ^k^ | 0.98 | | 1.11 | | 1 | | 0 | | 4 | 0.91 | 1.06 | | 1 | | | 0 | | 4 | 1.08 | 1.16 | | 1 | | | 0 | | 4 | 0.17 | 0.41 | | 0 | | | 0 | | 4 |
| Physical activity ^l^ |  | |  | |  | |  | |  |  |  | |  | | |  | |  |  |  | |  | | |  | |  |  |  | |  | | |  | |  |
| average last 3  months | 2.64 | | 1.30 | | 3 | | 1 | | 5 | 2.67 | 1.31 | | 3 | | | 1 | | 5 | 2.61 | 1.29 | | 2 | | | 1 | | 5 | 2.67 | 1.37 | | 2.5 | | | 1 | | 5 |
| last week | 2.41 | | 1.34 | | 2 | | 1 | | 5 | 2.40 | 1.33 | | 2 | | | 1 | | 5 | 2.42 | 1.35 | | 2 | | | 1 | | 5 | 2.33 | 1.63 | | 2 | | | 1 | | 5 |
| Personality ^m^ |  | |  | |  | |  | |  |  |  | |  | | |  | |  |  |  | |  | | |  | |  |  |  | |  | | |  | |  |
| openness | 3.32 | | 1.02 | | 3 | | 1 | | 5 | 3.40 | 0.98 | | 3 | | | 1 | | 5 | 3.23 | 1.06 | | 3 | | | 1 | | 5 | 3.5 | 1.05 | | 3.5 | | | 2 | | 5 |
| conscientious-  ness | 3.20 | | 0.86 | | 3 | | 1 | | 5 | 3.26 | 0.88 | | 3 | | | 1 | | 5 | 3.13 | 0.84 | | 3 | | | 1 | | 5 | 3.5 | 1.05 | | 3.5 | | | 2 | | 5 |
| extraversion | 2.64 | | 1.07 | | 3 | | 1 | | 5 | 2.80 | 1.05 | | 3 | | | 1 | | 5 | 2.48 | 1.08 | | 2 | | | 1 | | 5 | 1.83 | 0.75 | | 2 | | | 1 | | 3 |
| agree-  ableness | 2.62 | | 0.81 | | 3 | | 1 | | 5 | 2.53 | 0.82 | | 3 | | | 1 | | 5 | 2.73 | 0.80 | | 3 | | | 1 | | 5 | 2.5 | 0.55 | | 2.5 | | | 2 | | 3 |
| neuroticism | 2.93 | | 1.02 | | 3 | | 1 | | 5 | 3.12 | 1.00 | | 3 | | | 1 | | 5 | 2.71 | 1.01 | | 3 | | | 1 | | 5 | 3 | 1.10 | | 3 | | | 2 | | 5 |
| Loss of income | 0.25 | | 0.53 | | 0 | | 0 | | 2 | 2.23 | 0.53 | | 0 | | | 0 | | 2 | 0.28 | 0.54 | | 0 | | | 0 | | 2 | 0.17 | 0.41 | | 0 | | | 0 | | 2 |
|  | | | | *N (%)* | | | | | | *n (%)* | | | | | | | | | *n (%)* | | | | | | | | | *n (%)* | | | | | | | | |
| Working status | | | |  | | | | | |  | | | | | | | | |  | | | | | | | | |  | | | | | | | | |
| regular working hours | | | | 298 (61%) | | | | | | 154 (59%) | | | | | | | | | 140 (62%) | | | | | | | | | 4 (67%) | | | | | | | | |
| on short time | | | | 24 (5%) | | | | | | 10 (4%) | | | | | | | | | 14 (6%) | | | | | | | | |  | | | | | | | | |
| unability to pursue current occupa-  tion due to closed schools etc. | | | | 12 (2%) | | | | | | 9 (3%) | | | | | | | | | 3 (1%) | | | | | | | | |  | | | | | | | | |
| unability to pursue current occupa-  tion due to health protection  measures | | | | 12 (2%) | | | | | | 7 (3%) | | | | | | | | | 5 (2%) | | | | | | | | |  | | | | | | | | |
| in quarantine | | | | 13 (3%) | | | | | | 7 (3%) | | | | | | | | | 5 (2%) | | | | | | | | | 1 (17%) | | | | | | | | |
| sick leave (other than COVID-19) | | | | 17 (3%) | | | | | | 10 (4%) | | | | | | | | | 7 (3%) | | | | | | | | |  | | | | | | | | |
| job-seeking | | | | 31 (6%) | | | | | | 14 (5%) | | | | | | | | | 17 (8%) | | | | | | | | |  | | | | | | | | |
| housewife/ househusband | | | | 22 (4%) | | | | | | 20 (8%) | | | | | | | | | 2 (1%) | | | | | | | | |  | | | | | | | | |
| pensioner | | | | 61 (12%) | | | | | | 28 (11%) | | | | | | | | | 32 (14%) | | | | | | | | | 1 (17%) | | | | | | | | |
| Workplace | | | |  | | | | | |  | | | | | | | | |  | | | | | | | | |  | | | | | | | | |
| home office | | | | 180 (30%) | | | | | | 59 (32%) | | | | | | | | | 48 (29%) | | | | | | | | | 1 (25%) | | | | | | | | |
| regular workplace | | | | 195 (54%) | | | | | | 97 (52%) | | | | | | | | | 95 (57%) | | | | | | | | | 3 (75%) | | | | | | | | |
| partly home office. partly regular  workplace | | | | 55 (15%) | | | | | | 30 (16%) | | | | | | | | | 25 (15%) | | | | | | | | |  | | | | | | | | |
| Mental Disorder | | | |  | | | | | |  | | | | | | | | |  | | | | | | | | |  | | | | | | | | |
| no | | | | 271 (55%) | | | | | | 121 (47%) | | | | | | | | | 149 (66%) | | | | | | | | | 6 (100%) | | | | | | | | |
| yes | | | | 214 (44%) | | | | | | 136 (53%) | | | | | | | | | 73 (32%) | | | | | | | | |  | | | | | | | | |
| don’t know | | | | 5 (1%) | | | | | | 2 (1%) | | | | | | | | | 3 (1%) | | | | | | | | |  | | | | | | | | |
| COVID-19 | | | |  | | | | | |  | | | | | | | | |  | | | | | | | | |  | | | | | | | | |
| Tested positive | | | |  | | | | | |  | | | | | | | | |  | | | | | | | | |  | | | | | | | | |
| no | | | | 472 (96%) | | | | | | 247 (95%) | | | | | | | | | 219 (97%) | | | | | | | | | 6 (100%) | | | | | | | | |
| currently ill | | | | 9 (2%) | | | | | | 7 (3%) | | | | | | | | | 2 (1%) | | | | | | | | |  | | | | | | | | |
| recovered | | | | 9 (2%) | | | | | | 5 (2%) | | | | | | | | | 4 (2%) | | | | | | | | |  | | | | | | | | |
| Sick relatives | | | |  | | | | | |  | | | | | | | | |  | | | | | | | | |  | | | | | | | | |
| no | | | | 454 (93%) | | | | | | 233 (90%) | | | | | | | | | 215 (96%) | | | | | | | | | 6 (100%) | | | | | | | | |
| currently ill | | | | 8 (16%) | | | | | | 6 (2%) | | | | | | | | | 2 (1%) | | | | | | | | |  | | | | | | | | |
| recovered | | | | 28 (6%) | | | | | | 20 (8%) | | | | | | | | | 8 (4%) | | | | | | | | |  | | | | | | | | |
|  | | *No* | | *Yes* | | | | | | *No* | | | | | *Yes* | | | | *No* | | | | | *Yes* | | | | *No* | | | | | *Yes* | | | |
| Working within  health care system | | 414 (84%) | | 76 (16%) | | | | | | 210 (81%) | | | | | 49 (19%) | | | | 200 (89%) | | | | | 25 (11%) | | | | 4 (67%) | | | | | 2 (33%) | | | |
| Domestic violence | |  | |  | | | | | |  | | | | |  | | | |  | | | | |  | | | |  | | | | |  | | | |
| last week | | 485 (99%) | | 5 (1%) | | | | | | 257 (99%) | | | | | 2 (1%) | | | | 222 (99%) | | | | | 3 (1%) | | | | 6 (100%) | | | | |  | | | |
| in the last year  (except last week) | | 476 (97%) | | 14 (3%) | | | | | | 249 (96%) | | | | | 10 (4%) | | | | 221 (98%) | | | | | 4 (2%) | | | | 6 (100%) | | | | |  | | | |
| Chronic illness | | 229 (47%) | | 261 (53%) | | | | | | 118 (46%) | | | | | 141 (54%) | | | | 111 (49%) | | | | | 114 (51%) | | | | 6 (100%) | | | | |  | | | |
| COVID-19 | |  | |  | | | | | |  | | | | |  | | | |  | | | | |  | | | |  | | | | |  | | | |
| relatives lost | | 476 (97%) | | 14 (3%) | | | | | | 252 (97%) | | | | | 7 (3%) | | | | 218 (97%) | | | | | 7 (3%) | | | | 6 (100%) | | | | |  | | | |
| *Note*: ^a^ Average daily consumption in minutes measured via smartphone;  ^b^ Sum score of 2 items answered on a 5-point scale from 1 (“Always”) to 5 (“Never”);  ^c^ Sum score of 2 items answered on a 2-point scale from 0 (“No”) to 1 (“Yes”);  ^d^ 3-point scale from 0 (“Not bothered”) to 2 (“Bothered a lot”);  ^e^ Sum score of 3 items answered on a 5-point scale from 1 (“Very often”) to 5 (“Never”);  ^f^ 5-point scale from 0 (“Very bad”) to 4 (“Very good”);  ^g^ 3-point scale from 0 (“Yes, it got worse”) to 2 (“Yes, it has improved”);  ^h^ Sum score of 11 items answered on a 3-point scale from 1 (“Not bothered”) to 5 (“Bothered a lot”);  ^i^ 5-point scale from 1 (“Very good”) to 5 (“Very bad”);  ^j^ Sum scores answered on a 4-point scale from 1 (“I haven't been doing this at all”), 2 (“I've been doing this a little bit”), 3 (“I've been doing this a medium amount”), 4 (“I've been doing this a lot”);  ^k^ 5-point scale from 0 (“None”) to 4 (“Six to seven times a week”);  ^l^ 5-point scale from 1 (“No sporting activity”) to 5 (“Regularly more than 4 hours a week”);  ^m^ Sum scores of 2 items answered on a 5-point scale from 1 (“Disagree strongly”) to 5 (“Agree strongly”). | | | | | | | | | | | | | | | | | | | | | | | | | | | | | | | | | | | | |
